# Neuroinflammation: The Pathogenic Mechanism of Neurological Disorders

**DOI:** 10.3390/ijms23105744

**Published:** 2022-05-20

**Authors:** Ali Gorji

**Affiliations:** 1 Epilepsy Research Center, Westfälische Wilhelms-Universität, 48149 Münster, Germany; gorjial@uni-muenster.de; Tel.: +49-(251)-835-5564; 2 Department of Neurosurgery and Neurology, Westfälische Wilhelms-Universität, 48149 Münster, Germany; 3 Department of Neurology and Institute of Translational Neurology, Westfälische Wilhelms-Universität, 48149 Münster, Germany; 4 Shefa Neuroscience Research Center, Khatam Alanbia Hospital, Tehran 1996835911, Iran; 5 Neuroscience Research Center, Mashhad University of Medical Sciences, Mashhad 9177948564, Iran

Neuroinflammation is implicated in the pathophysiology of several neurological diseases. The key role of neuroinflammation in a wide range of neurological disorders makes it highly attractive for diagnostic examinations and therapeutic interventions in recent years [1]. The objective of this Special Issue is to collect some of the latest efforts in the field of neuroinflammation to discover the underlying pathophysiological mechanisms as well as the diagnostic and therapeutic approaches to various human diseases. Neuroinflammation is the innate and adaptive immune responses that are initiated toward a variety of harmful insults (such as infection, ischemia, stress, and trauma) through the release of inflammatory mediators (such as cytokines, chemokines, and reactive oxygen species) by various immune cells (like microglia, astrocytes, peripherally derived immune cells, and endothelial cells) [2]. Neuroinflammation in the initial stage is mainly beneficial and protective; however, evidence from both clinical and experimental studies indicates that prolonged or excessive inflammation is a pivotal pathological driver of several neurological disorders, such as cerebrovascular diseases (CVD), traumatic brain and spinal cord injuries, neurodegenerative diseases, epilepsy, multiple sclerosis (MS), psychological disorders, and chronic pain. Neuroinflammation is the common mechanism that connects ischemic, degenerative, traumatic, demyelinating, epileptic, and psychiatric pathologies [1,2,3]. 

Both experimental and clinical investigations suggest the essential role of neuroinflammation in medically intractable epilepsy [4]. Several intrinsic factors (such as intensity of injury and genetic factors) and/or environmental factors (like infections and stress) could modulate the strength and persistence of the inflammatory processes following acute brain insult and contribute to the process of epileptogenesis [5]. Among the various factors, the role of stress-induced neuroinflammation in epileptogenesis received less attention. Evidence indicates that repeated exposure to repetitive acute stress, particularly in early life, alters the function of the hypothalamic-pituitary-adrenal axis, exacerbates hippocampal sclerosis, and enhances vulnerability to epileptogenesis [6,7]. The stress-induced neuroinflammation in association with the dysfunction of various neurotransmitters, glucocorticoid receptors, and different neurotrophic factors could aggravate the established pro-inflammatory effects of seizures and lead to the enhancement of neuronal network excitability [8,9]. Based on the evidence of a potential correlation between stress-induced inflammation and epilepsy, Espinosa-Garcia and colleagues have described the importance of early interventions for both acute and chronic stress in the improvement of diagnosis, therapy, and outcomes for patients with epilepsy, particularly for subjects with psychiatric comorbidities [10]. Moreover, modulation of inflammatory processes and mediators represent relevant potential targets for the treatment of epilepsy [11]. Interleukin-1β (IL-1β), a pro-inflammatory cytokine that activates various cytokine cascades and enhances seizure susceptibility, is a potential therapeutic target for medically intractable epilepsy [12,13]. Assessment of patients with febrile infection-related epilepsy syndrome indicates the potential role of neuroinflammation secondary to a functional deficiency in the endogenous interleukin-1 receptor antagonist in developing drug-resistant epilepsy [14]. Yamanaka et al., in this issue, evaluated the potential role of anakinra, an interleukin-1 receptor antagonist, in the treatment of patients with febrile infection-related epilepsy syndrome and suggested prospective investigations to understand the optimal timing, dosage, and duration of anakinra application for prevention and treatment of this epilepsy syndrome [15].

Multiple lines of evidence suggest that neuroinflammation plays a pivotal role in the pathogenesis of CVD and it has been considered a potential target for therapeutic intervention [16]. Neuroinflammation exerts both beneficial and detrimental effects on CVD, which should be considered for designing novel therapeutic strategies [17]. Among various factors, microRNAs (miR), small conserved non-coding single-stranded RNA, play a modulatory role in CVD-induced neuroinflammation [18]. In this issue, Kashif et al. described a comprehensive evaluation of the role of different miR in CVD. Their study concluded that several miR, such as miR-7, miR-23b, miR-223, miR-132, and miR-194-5p may exert a neuroprotective effect, whereas other miR, like miR-222 and miR-494, could exhibit a detrimental effect in CVD [19]. They have suggested that appropriate modulation of miR expression could be a therapeutic strategy to alleviate post-CVD neuroinflammation [19]. Furthermore, electrical stimulation of distinct brain regions has been suggested as an approach that alleviates the inflammatory processes after brain ischemic insults [20,21]. However, the exact mechanism of action still needs to be elucidated. In this issue, Schuhmann et al. investigated the effect of high-frequency stimulation of the mesencephalic locomotor region on photothrombotic stroke-induced neuroinflammation in rats. They have shown that continuous stimulation of the mesencephalic locomotor area led to a significant reduction in the values of various inflammatory mediators, such as IFN-γ, TNF-α, and IL-1α, within the perilesional region, possibly by the modulation of the cholinergic anti-inflammatory pathway [22].

Toll-like receptors (TLR), a family of evolutionarily transmembrane proteins and a pivotal part of the innate immune system, are key components of inflammatory processes in different neurological disorders, including CVD and neurodegenerative diseases [23]. In this issue, Ashayeri Ahmadabad et al. discussed the key role of the modulation of different Toll-like receptors and downstream signaling pathways on various CVD. Considering the beneficial effects of the activation of Toll-like receptors in the initial phase and detrimental in the late stage of cerebrovascular diseases, they suggested that appropriate modulation of TLR could be a promising approach for the prevention and treatment of CVD [24]. Furthermore, several in vitro and in vivo experiments revealed the crucial role of various TLR, such as TL4 and TLR7, in neuronal damage and cell death as well as in triggering neurodegenerative processes [25,26]. In this issue, Qin et al. revealed that the occurrence of apoptotic neuronal death in an animal model of progressive neurodegeneration was associated with the enhancement of TLR7 expression as well as tumor necrosis factor-related apoptosis-inducing ligand (TRAIL) receptors. The application of TRAIL-neutralizing monoclonal antibodies in this experimental model inhibited neuronal death; suggesting TRAIL as a potential therapeutic target to slow neurodegenerative processes [27].

Regulation of TLR can also modulate the impact of spreading depolarization (SD) [28], a negative propagating neuroglial wave implicated in various neurological disorders, including brain trauma [3]. In this issue, Aboghazale and colleagues evaluated the electrophysiological alterations of rat brain after traumatic brain injury and have shown the occurrence of both SD and SD-induced depression of cortical activity. Moreover, their findings revealed that while the occurrence of SD following closed brain trauma led to enhanced oxidative stress (elevated reactive oxygen species), traumatic brains exhibited a decreased antioxidant defense (downregulation of mRNA expression of antioxidant enzymes in response to oxidative stress) [29]. In addition to traumatic brain injury, inflammatory processes play a regulatory role in various mechanisms that result in tissue repair, damage, and/or death following the spinal cord injury (SCI) [30]. In the present issue, Brockie et al., have described the complex interaction of microglia with neurons, astrocytes, infiltrating monocytes, and endothelial cells to maintain the inflammatory processes following SCI. the crosstalk between microglia with other cells is essential to initiate both acute and chronic inflammatory cascades following SCI. It has been suggested that appropriate modulation of microglia activation in both SCI-induced acute and chronic inflammation could be a therapeutic target to promote their regenerative capabilities and improve functional recovery after SCI [31].

Furthermore, the clinical and experimental findings support the pathogenic role of neuroinflammation in various psychiatric disorders [32]. Psychosocial stress, early life adversity, and infection could lead to pro-inflammatory activation of microglia, which is associated with increased cytokine values, cyclooxygenase activation, dysregulation of neurotransmitters, enhanced oxidative stress, impairment of blood–brain-barrier function, and cognitive dysfunction [33]. In the present issue, Vojtechova et al. have investigated the effects of maternal immune activation on the brain and behavior of adolescent and adult offspring. Their findings indicate gender-specific chronic inflammation-induced behavioral changes, whereas alterations in the brain structures observed in young rats are sex-independent. Their data suggest the impact of gender-specific immunity on neuroinflammation-induced cognitive and behavior changes [34]. 

The cyclic GMP–AMP synthase–stimulator of interferon genes (STING) pathway plays a pivotal role in coupling the sensing of DNA to the induction of strong innate immune responses [35]. Multiple lines of evidence indicate the implication of the STING pathway in both acute and chronic inflammatory conditions associated with different neurological disorders, including MS [36,37]. Using an animal model of MS, Masanneck et al. are the first to provide evidence that STING values decreased in the peripheral lymphoid tissue, while its levels significantly enhanced within the central nervous system throughout the disease. Furthermore, they demonstrated a similar pattern in human peripheral immune cells during the acute phases of relapse-remitting MS compared with remitting phases and healthy subjects. This study suggests the modulation of STING as a potential approach for the reduction of neuroinflammation and improving outcomes in patients with MS [38].

More efforts are required to elucidate the complex interplay among different factors and pathways that contribute to inflammatory processes in diverse central nervous system disorders. The modulation of the immune system in specific manners can provide an opportunity to treat several of these diseases with similar approaches and improve the outcomes [1,39]. Therapeutic strategies targeting neuroinflammation are responsible for some of the novel recent advances in the field of neurology [40].

## Abbreviations

CVD	Cerebrovascular diseases
IL-1β	Interleukin-1β
miR	microRNAs
MS	Multiple sclerosis
SCI	Spinal cord injury
SD	Spreading depolarization
STING	Stimulator of interferon genes
TLR	Toll-like receptors
TRAIL	Tumor necrosis factor-related apoptosis-inducing ligand
